# Modelling menstrual cycle length in athletes using state-space models

**DOI:** 10.1038/s41598-021-95960-1

**Published:** 2021-08-20

**Authors:** Thiago de Paula Oliveira, Georgie Bruinvels, Charles R Pedlar, Brian Moore, John Newell

**Affiliations:** 1 School of Mathematics, Statistics and Applied Mathematics, National University of Ireland, Galway, Ireland; 2 Orreco, Business Innovation Centre, National University of Ireland, Galway, Ireland; 3 The Insight Centre for Data Analytics, National University of Ireland, Galway, Ireland; 4 St Mary’s University, Twickenham, UK

**Keywords:** Statistical methods, Statistics

## Abstract

The ability to predict an individual’s menstrual cycle length to a high degree of precision could help female athletes to track their period and tailor their training and nutrition correspondingly. Such individualisation is possible and necessary, given the known inter-individual variation in cycle length. To achieve this, a hybrid predictive model was built using data on 16,524 cycles collected from a sample of 2125 women (mean age 34.38 years, range 18.00–47.10, number of menstrual cycles ranging from 4 to 53). A mixed-effect state-space model was fitted to capture the within-subject temporal correlation, incorporating a Bayesian approach for process forecasting to predict the duration (in days) of the next menstrual cycle. The modelling procedure was split into three steps (1) a time trend component using a random walk with an overdispersion parameter, (2) an autocorrelation component using an autoregressive moving-average model, and (3) a linear predictor to account for covariates (e.g. injury, stomach cramps, training intensity). The inclusion of an overdispersion parameter suggested that $26.36\%$ $[23.68\%,29.17\%]$ of cycles in the sample were overdispersed. The random walk standard deviation for a non-overdispersed cycle is $27.41 \pm 1.05$ [1.00, 1.09] days while under an overdispersed cycle, the menstrual cycle variance increase in 4.78 [4.57, 5.00] days. To assess the performance and prediction accuracy of the model, each woman’s last observation was used as test data. The root mean square error (RMSE), concordance correlation coefficient and Pearson correlation coefficient (r) between the observed and predicted values were calculated. The model had an RMSE of 1.6412 days, a precision of 0.7361 and overall accuracy of 0.9871. In conclusion, the hybrid model presented here is a helpful approach for predicting menstrual cycle length, which in turn can be used to support female athlete wellness.

## Introduction

The availability of mobile apps developed to track the menstrual cycle is growing as they are becoming increasingly popular for contraception purposes, fertility awareness and exercise planning. These apps can be grouped broadly as calendar-based, basal body temperature (BBT), or symptothermal^1–3^. Calendar apps generally use simple algorithms based on empirical measurements to predict cycle phase length^4^; BBT apps describe a woman’s menstrual variation through her basal body temperature rise^5^ and symptothermal apps measure parameters such as cervical mucus changes, bleeding period and so on^2^.

The mobile app that generated the data used in the study is called FitrWoman. It is a free calendar-based app that enables users to track their menstrual cycle and symptoms, and provides relevant information about wellness, nutrition and exercise, based on the athlete’s predicted menstrual cycle phases and length. The user inputs daily information on 25 symptom variables such as flow, bloating, constipation, injury, illness, irritability and weakness. The target audience is female athletes who wish to track their menstrual cycle to improve their performance and understanding of their individual cycle.

As a woman’s body may respond and adapt differently throughout their cycle, different planning and preparation over the menstrual cycle phases^6–8^ might be required. McNulty et al.^9^ observed through meta-analysis that exercise performance might be trivially reduced during the early follicular phase of the menstrual cycle when compared to the other phases.

As few apps are accurate in terms of menstrual cycle length prediction^10^, the development of an appropriate, exact parametric model for one-step-ahead forecast cycle length is required. Such a model should take into account the between and within-woman variability to identify menstrual cycle patterns and how each symptom could affect cycle length, alongside the implications of significant alterations in cycle length.

According to several studies^11–14^, the menstrual cycle length can be classified into two groups ‘standard‘ and ‘menstrual dysfunction‘, where a cycle length greater than 35 days is classified as ‘menstrual dysfunction‘ and otherwise as standard. Many statistical models have been proposed in the literature to describe these different groups of menstrual cycles^2,15–18^. Generally, cycle length related to the ‘standard‘ group can be analysed using classical statistical approaches. In contrast, the mixture of standard and non-standard cycles can be analysed using a mixture distribution accounting for the significant symmetric distribution and the component corresponding to the heavy right tail^14,15^. To account for the within-individual variability, we focused on the dynamic aspect of menstrual cycles over time, as discussed by Bortot et al. (2010)^16^, who derived a predictive distribution based on individual repeated measurements using a state-space model formulation. According to these authors^16^, state-space models under a Bayesian approach have the advantage of incorporating between subject information to compensate for the relatively large number of subjects with a low quantity of repeated measurements and to make predictions for women not included in the sample.

It is well-established that having a regular menstrual cycle is a ’vital sign‘ demonstrating that the body is likely to be in an adaptive state and is tolerating the physical and psychological stressors that are being placed on it^19^. Significant elongations in cycle length are associated with adverse health and fertility outcomes^20–23^, therefore gaining a better understanding of the interrelating risk factors for cycle length extension is important.

In this paper, the first objective was to develop an appropriate parametric state-space formulation for the marginal distribution of standard menstrual cycles for female athletes. In addition, symptom variables were included in the model’s linear predictor to evaluate how the individual reported symptoms might affect an athlete’s menstrual cycle duration. The second aim was to develop a one-step-ahead forecasting interval approach, based on a state-space formulation, to describe the experimental and state process while considering both between and within-woman variability.

## Results and discussion

Results from the state-space models, state-space mixed-effects models and linear mixed-effects models (LMM), fitted using the available data, are summarised in Table 1. In general, the Bayesian information criteria (BIC) suggests that the random walk models fitted better than the LMM when modelling menstrual cycle length, in agreement with the results reported by Bortot at al. (2010)^16^ while contradicting the results of^2^ who report an $R^2=0.99$ when fitting a simple linear regression.

**Figure 1:**
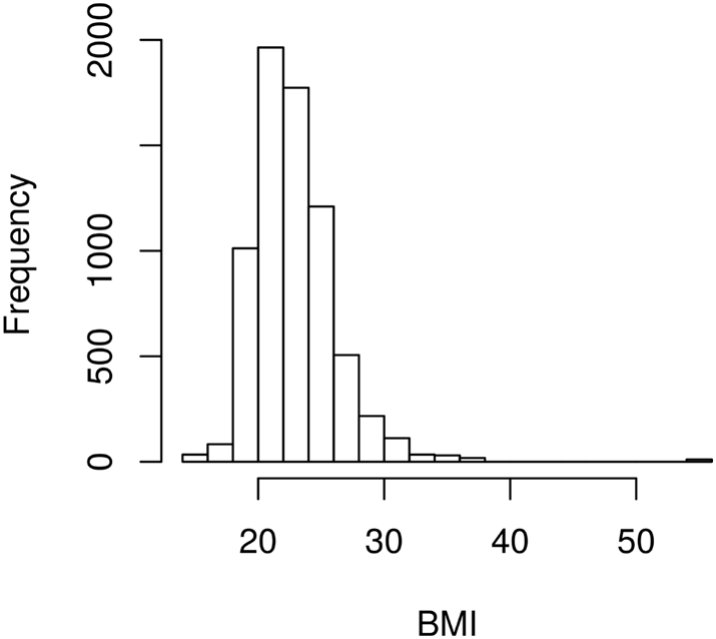
Histogram of Body Mass Index (BMI) classification.

The inclusion of $r_{ij}$ to model overdispersed cycle lengths was fundamental to describe menstrual cycle dynamics as evidenced by the BIC criteria where a reduction of $56.22\%$ compared to $y_{ij}=m_{ij}+\epsilon _{ij}$, and $56.62\%$ compared to $y_{ij}=\beta _0+b_{0i}+\left( \beta _{1}+b_{1i}\right) Age_{ij}+\epsilon _{ij}$ is evident, as shown in Fig. 1. Additionally, the inclusion of a moving average (MA) parameter was necessary to capture the dynamism of shorter cycles followed by longer cycles and vice-versa. In summary, a random walk with a random variable to capture overdispersion $r_{ij}$ plus a MA(1) model demonstrated the best fit to the data.

**Table 1:** Model selection criteria for stages I and II; number of parameters (N. Par.), root mean square error (RMSE), concordance correlation coefficient (CCC), Pearson correlation coefficient (r) between fitted and predicted test data, and Bayesian information criterion (BIC).

Model	N. Par.	Forecasting			BIC
		RMSE	CCC	r	
$y_{ij}=m_{ij}+\epsilon _{ij}$	3	1.6066	0.7327	0.7537	16,886.70
$y_{ij}=m_{ij}+\text{ AR(1) }$	4	1.5956	0.7251	0.7546	17,920.96
$y_{ij}=m_{ij}+\text{ MA(1) }$	4	1.6108	0.7348	0.7533	17,694.21
$y_{ij}=m_{ij}+\text{ ARMA(1,1) }$	5	1.5808	0.7360	0.7603	17,695.83
$y_{ij}=m_{ij}+r_{ij}+\epsilon _{ij}$	5	1.6449	0.7131	0.7283	7393.54
$y_{ij}=m_{ij}+r_{ij}+\text{ AR(1) }$	6	1.6332	0.7136	0.7323	8413.30
$y_{ij}=m_{ij}+r_{ij}+\text{ MA(1) }$	6	1.6412	0.7266	0.7361	7381.61
$y_{ij}=m_{ij}+r_{ij}+\text{ ARMA(1,1) }$	7	1.6255	0.7203	0.7363	7460.37
$y_{ij}=\beta _0+b_{0i}+\left( \beta _{1}+b_{1i}\right) Age_{ij}+\epsilon _{ij}$	5	1.6274	0.7257	0.7457	17,042.26
$y_{ij}=\beta _0+b_{0i}+\left( \beta _{1}+b_{1i}\right) Age_{ij}+\text{ AR(1) }$	6	1.6640	0.7205	0.7374	17,413.73
$y_{ij}=\beta _0+b_{0i}+\left( \beta _{1}+b_{1i}\right) Age_{ij}+\text{ MA(1) }$	6	1.6810	0.7171	0.7326	17,305.01
$y_{ij}=\beta _0+b_{0i}+\left( \beta _{1}+b_{1i}\right) Age_{ij}+\text{ ARMA(1,1) }$	8	1.6832	0.7164	0.7320	17,314.24

To assess model performance, we compared the forecasts of these models using the RMSE of one-step-ahead predictions, CCC and Pearson correlation coefficient evaluated on the test group. Table 1 demonstrates that better forecast predictions were made using a random walk rather than an LMM and that there was little difference between the random walk models in terms of forecasting. As a consequence, the BIC criteria can be used to select the error structure. After selecting the trend and error structures, the next stage of the analysis was the selection of potentially useful explanatory variables. The set of 28 available represented a variety of reported symptoms by the *i*-th woman, including an interval-based variable representing a woman’s body mass index (Kg/$\hbox {m}^2$) (Table 2), classified as discussed by Corbel at al. (2004)^24^. In this analysis, underweight classes I and II were classified as ’severely’ and ’very severely underweight’ while obese classes I, II, and III represented moderately, severely and very severely obese, respectively. The sample of women had a reported BMI of between 14.44 and 54.25, with a mean of 22.85 Kg/$\hbox {m}^2$; the absolute frequency is shown as a histogram in Fig. 1.

**Table 2:** Histogram of BMI and body mass index (BMI) classification.

Category	BMI $\left( \text{ Kg }/\text{m}^2\right)$	
	From	To
Underweight II		15
Underweight I	$> 15$	16
Underweight	$> 16$	18.5
Normal	$> 18.5$	25
Overweight	$> 25$	30
Obese Class I	$> 30$	35
Obese Class II	$> 35$	40
Obese Class III	$> 40$	

The selected state-space model summary with posterior means and 95% credibility intervals for the population parameters (after predictor selection) is presented in Table 3.As the model parameterisation facilitates the interpretation of the role played by the explanatory variables, our analysis reveals important insights on how some symptoms affect menstrual cycle length.

**Table 3:** Posterior means and 95% Bayesian credibility interval for $\varvec{\Theta }$.

Parameter	Estimate	SE	95% Credible Interval	
			Lower	Upper
$\beta _0$	27.4141	0.0440	27.3283	27.4996
$\pi$	0.2636	0.0142	0.2368	0.2917
$\theta _0$	− 0.0915	0.3160	− 0.1563	− 0.0320
$\alpha _1$ (Injury)	0.2965	0.1038	0.0554	0.4768
$\alpha _2$ (Stomach Cramps)	0.1682	0.0585	0.0567	0.2835
$\alpha _3$ (Tender Breasts)	− 0.1540	0.0457	− 0.2443	− 0.0624
$\alpha _4$ (Flow Amount: Heavy)	− 0.0816	0.0861	− 0.2492	0.0882
$\alpha _5$ (Flow Amount: Medium)	0.0290	0.0196	− 0.0094	0.0675
$\alpha _6$ (Flow Amount: Light)	− 0.1320	0.0560	− 0.2414	− 0.0239
$\alpha _{7}$ (Flow Amount: Spotting)	0.0589	0.0712	− 0.0792	0.2012
$\alpha _{8}$ (Flow Amount: None)	0.0093	0.0208	− 0.0314	0.0492
$\sigma _{\eta }$	1.0417	0.0231	0.9971	1.0875
$\sigma _{w}$	4.7803	0.1096	4.5738	5.0007
$\sigma _{\epsilon }$	1.5407	0.0449	1.4504	1.6259

We found that the overall menstrual cycle length without any reported symptoms was around $27.41 \left[ 27.33,27.50\right]$ days, which is in agreement with Guo et al (2006)^15^ and Bull et al. (2019)^2^. Additionally, the reporting of injury, stomach cramps and flow amount was associated with increased menstrual cycle length. In contrast, the reporting of tender breasts was associated with decreased cycle length. For example, if a woman reported tender breasts ten times over her cycle, as a consequence, her predicted menstrual cycle length is estimated to reduce, on average, by $0.154 \times 10 = 1.54$ days.

Self-track symptoms quality depends on both user engagement, app design and unambiguous language to describe the level of a symptom. Consequently, to make it more consistent, filtering the original database based on the scientific literature is a critical way to reduce bias in the covariates used to fit the model, as described by Li et al. (2020)^14^.

**Figure 2:**
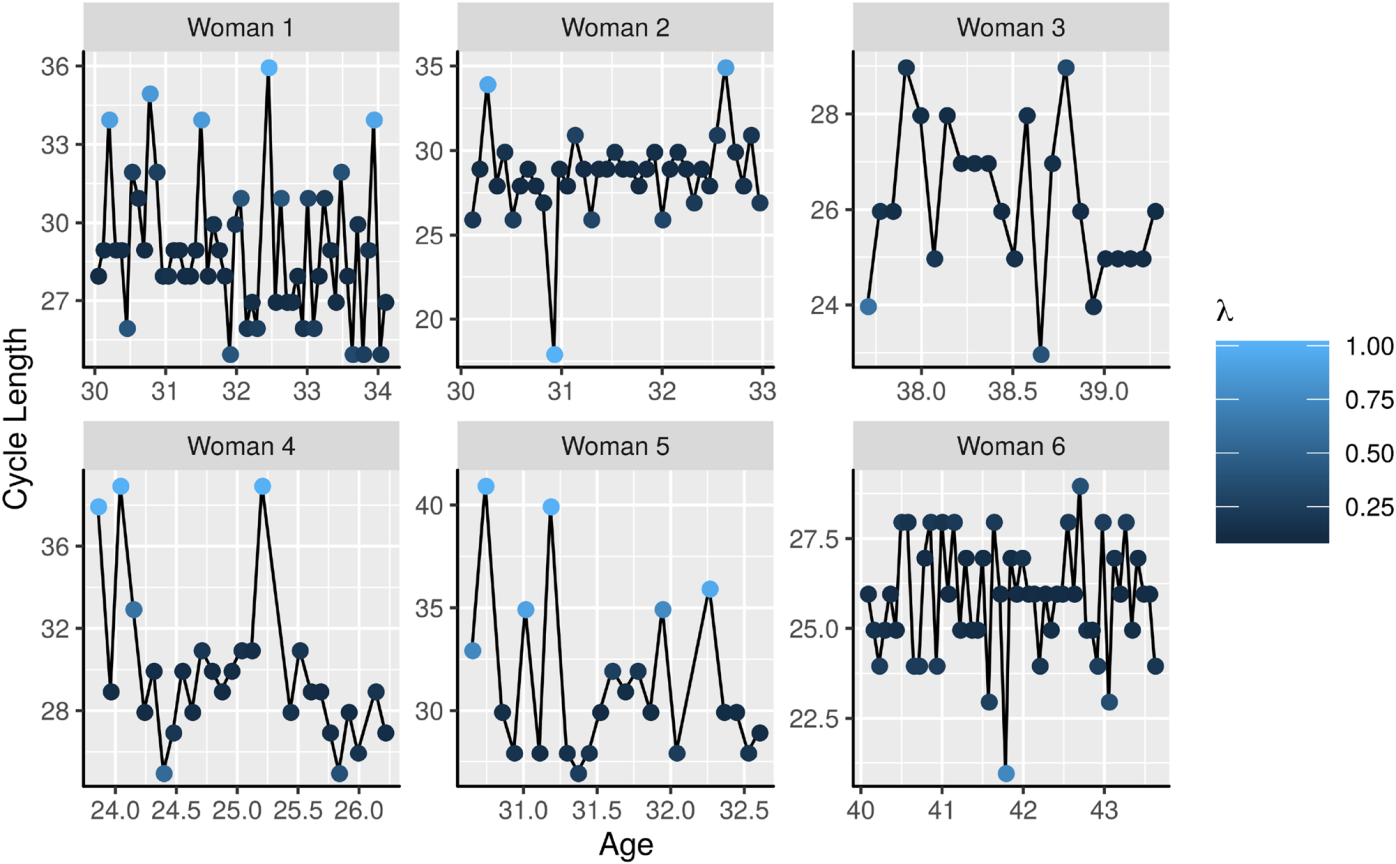
Example of six women profiles showing the probability that the proposed model considers an observation as overdispersed, where $\lambda$ represents the probability of $\lambda _{ij}$ being equal 1 for a given observation.

The estimated value of $\pi$ suggests that the probability of a non-standard (overdispersed) menstrual cycle length occurring in this population of interest is 0.2636. Consequently, we can infer that $26.36\%$ $[23.68\%,29.17\%]$ of cycles in the sample are overdispersed. Furthermore, while a non-overdispersed cycle had a standard deviation (SD) of $\sigma _{\eta }=1.0417$ [0.9971, 1.0875], the SD of an overdispersed cycle increases where $\sigma _{w} = 4.7803$ [4.5738, 5.0007], which represents a 4-fold increment. According to Najmabadi et al. (2020)^25^, between and within-variability in cycle characteristics should be emphasised as an important health indicator to assess behavioural, metabolic, and environmental factors. Therefore, the inclusion of $\theta$ and $\sigma _{w}$ play an essential role in the proposed model, as illustrated in Fig. 2. This Figure shows the probability that the proposed model (3) considers an observation as overdispersed where the results clearly demonstrate that $r_{ij}=\lambda _{ij}w_{ij}$ is capturing menstrual cycles with overdispersion.

Using this model, knowledge and understanding can be gleaned as to how symptom variables affect the menstrual cycle, which is essential for individual athletes, coaches and healthcare professionals. Furthermore, these results can improve the forecasting intervals, helping women to know more about their bodies and cycles based on symptoms during a particular phase of their cycles. Further work is needed to translate these findings into recommendations. Although information relating to follicular and luteal phases was not available in the data, a strong linear correlation between menstrual cycle length and follicular phase has been reported^26–28^. Where the correlation tended to increase with age. To predict ovulation time, further studies, which include both luteal and follicular phases and basal body temperature (BBT), are needed to extend the proposed model^2^.

Although an ARMA(1,1) model was not needed in this analysis, we have demonstrated that some women have a positive lag-one autocorrelation while others have a negative lag-one autocorrelation. These results contradict the findings of^16,29^ who report a small general negative autocorrelation for a woman’s profile. In order to better investigate the variability of an autoregressive coefficient, we modified the state-space formulation to accommodate this source of random variation by assuming that $\phi _{i}=\phi _{0}+\phi _{0i}$, with $\phi _{0i} \sim N\left( 0,\sigma ^2_{\phi }\right)$. However, the normality assumption for $\phi _{0i}$ was not justified as the normal Q-Q plot suggested a distribution with heavy tails and asymmetry; as a consequence, $80\%$ of points were outside of the 95% simulated envelopes for this random effect (Figure S1).

We also observed that some women had a long cycle followed by a short cycle and vice versa, as observed by Bortot et al. (2010)^16^. However, we found while ${\hat{\theta }}=-0.0915$ with $\text{ CI}_{95\%}: \left[ -0.1563, -0.0320\right]$ the estimate of the same parameter described by Bortot et al. (2010)^16^ was $-0.61$ $[-0.77,-0.45]$. It appears that the sample of female athletes that these analyses are based on had more regular menstrual cycles than a sample of 1,798 women observed from clients of the Catholic Marriage Advisory Council of England and Wales. Although we have a higher number of women in our sample than in^16^, the time series in their sample were longer (up to 109 measurements) compared with up to 55 measurements in this sample. In order to account for the between-subject variability, we included a random effect in the moving-average coefficient given by $\theta _{i}=\theta _{0}+\theta _{0i}$, with $\theta _{0i} \sim N\left( 0,\sigma ^2_{\theta }\right)$. However, we observed the same problem as reported when considering the autoregressive coefficient where more than $70\%$ of points were outside of the 95% simulated envelopes, lower asymmetry compared with $\phi _{0i}$ and heavy tails (Figure S2). Therefore, to avoid bias in individual forecasting predictions, these random effects were dropped from the model. Further work is needed to accommodate individual estimation for the autocorrelation and moving-average coefficients to improve model performance at the individual level.

**Figure 3:**
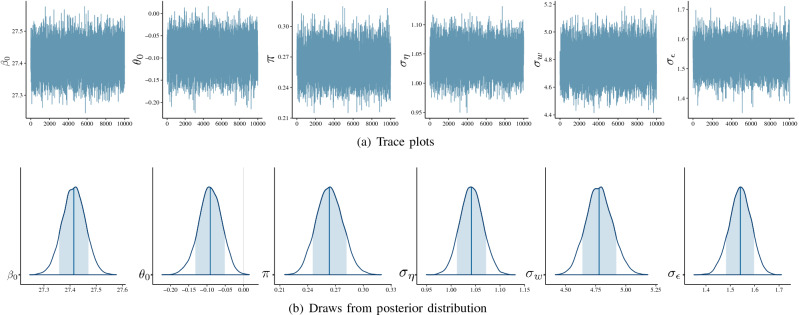
(**a**) Trace plots of Markov chains and (**b**) Markov chain Monte Carlo (MCMC) draws from the posterior distribution of the parameters $\beta _0$, $\theta _0$, $\pi$, $\sigma _{\eta }$, $\sigma _{w}$, and $\sigma _{\epsilon }$ based on a sample of length 3000.

**Figure 4:**
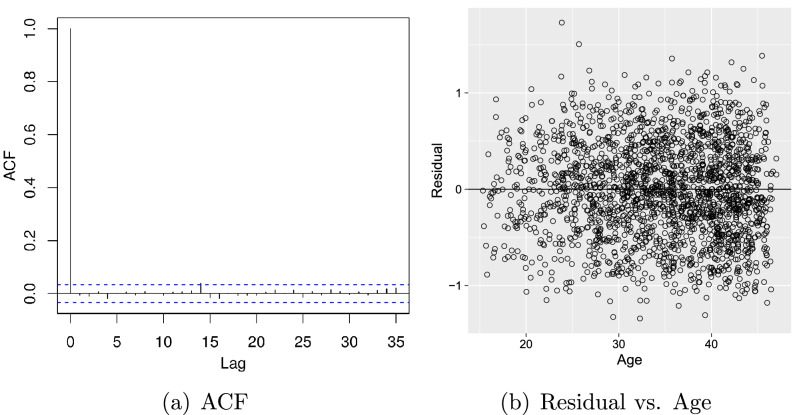
(**a**) Residual autocorrelation plot, and (**b**) residual versus age.

The analysis workflow was as follows: we initially checked the Bayesian assumptions and the posterior distribution using suitable plots of the Markov Chain Monte Carlo (MCMC) draws from the posterior distribution and Gelman-Rubin diagnostic and autocorrelation plots of all model parameters. Figure 3a shows the iterates of $\beta _0$, $\pi$, $\theta _0$, $\sigma _{\eta }$, $\sigma _{w}$, and $\sigma _{\epsilon }$ after a burn-in of 10,000 simulated iterations, which indicates convergence of the chains and stationary distributions, as the samples appear to be randomly sampled from the same region of the y-axis rarely venturing outside that area. The autocorrelation and Gelman-Rubin statistics^30^ were used to assess model convergence. The results suggest that the autocorrelation does not drop dramatically from lag 0 to 50 (Figure S3), indicating a moderate to high autocorrelation among samples. To reduce the impact of this problem, we stipulated a thinning of 50. On the other hand, the Gelman-Rubin statistic based on three chains showed all upper 95% confidence intervals were exactly equal to 1, meaning the chains had converged. Figure 3b shows the posterior densities obtained for estimated parameters derived from 3 Markov chains with 3000 samples per chain, leading to a computational time of around 23 hours executed on Dell Inspiron 17 7000 with 10$^{ \text{ th }}$ Generation Intel$\circledR$ $\hbox {Core}^{\text{ TM }}$ i7 processor, 1.80GHz $\times$ four-processor speed, 16GB random access memory (RAM) plus 20GB of swap space, 64-bit integers, and the platform used is a Linux Mint 19.2 Cinnamon system version 5.2.2-050202-generic. In summary, the posterior distribution has been well characterised by the drawn samples as no unexpected peaks or strange shapes in the posterior density were observed that could signify poor model convergence. As a final assessment, the autocorrelation function, as well as the standardized residual against the athlete’s age, were checked (Fig. 4). No serious discrepancies nor patterns that warrant attention were observed in both graphs.

**Figure 5:**
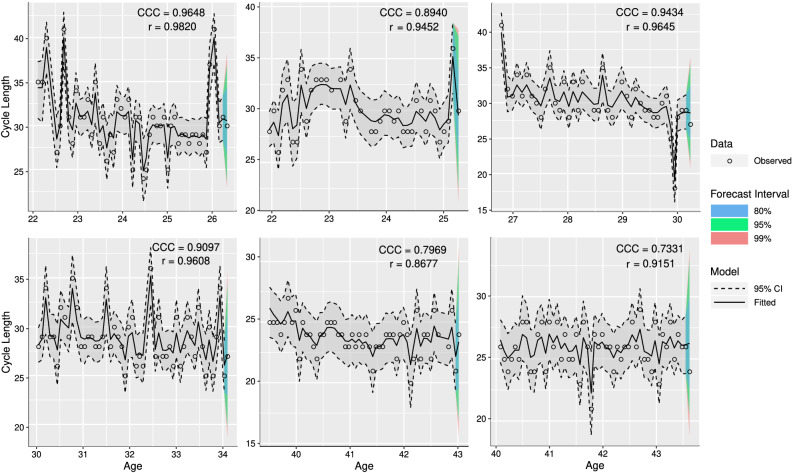
Age versus fitted menstrual cycle length for six women with more than 40 repeated measurements with addition of 95% credible interval (dashed line), 80%, 95%, and 99% forecast intervals for the next cycle, and observed menstrual cycle length as points. The estimated concordance correlation coefficient (CCC), and Pearson correlation coefficient (r) between fitted and observed values are described for each woman. Accuracy ($C_b$) can be obtained using $\text{ C}_{\text{ b }}=\text{ CCC }/\text{r }$.

Once the assumptions were verified, we evaluated the agreement between the fitted and observed values and forecast intervals. Figure 5 shows the fitted curves for menstrual cycle length of six women, their 95% credible interval, and the one-step-ahead point forecast with 80%, 95% and 99% forecast intervals. We observed that the random walk with overdispersion parameter and MA(1) model performed well in describing the complex dynamics of menstrual cycle length over time. This conclusion is underpinned by CCC’s residual diagnostic and high values and Pearson correlation between fitted and observed values by the woman. These results also show that linear or linear mixed-effects models should not be applied to explain the variability of menstrual cycle length. They generally do not follow the necessary assumptions of linearity–however, a study done in 2019 by Bull et al. (2019)^2^ appears to use linear models to explain cycle length observed from an extensive database of cycles collected through an app. The authors show an $R^2=0.987$ without any discussion as to whether the model assumptions are likely to be fulfilled; a high $R^2$ value does not necessarily imply that a regression model provided an adequate fit to the data^31^.

**Figure 6:**
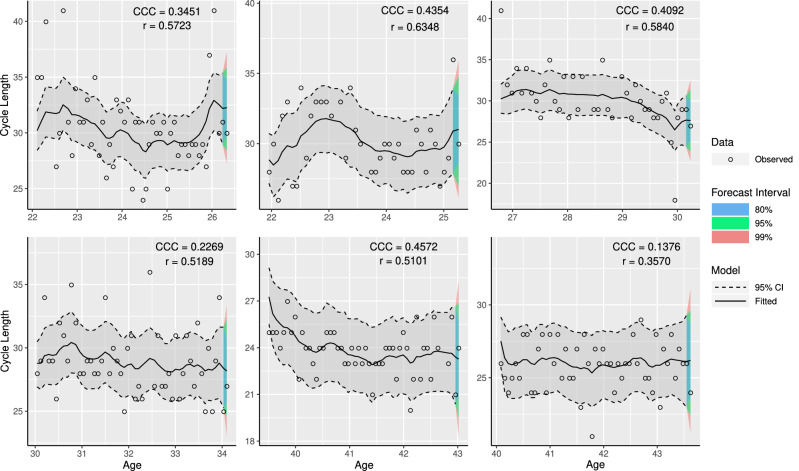
Age versus fitted menstrual cycle length for six women with more than 40 repeated measurements with addition of 95% credible interval (dashed line), 80%, 95%, and 99% forecast intervals for their next cycle, and observed menstrual cycle length (points) using the model $y_{ij}=m_{ij}+\gamma _{ij}+c_{ij}$, when dropping the term $r_{ij}$ from the model. The estimated concordance correlation coefficient (CCC), and Pearson correlation coefficient (r) between fitted and observed values are reported for each woman. Accuracy ($C_b$) can be obtained using $\text{ C}_{\text{ b }}=\text{ CCC }/\text{r }$.

The necessity of including $r_{ij}=\lambda _{ij}w_{ij}$ in our model to describe cycle length is demonstrated in Fig. 6 where the improvement in the point estimates, credible and forecasting intervals when $r_{ij}=\lambda _{ij}w_{ij}$ was and was not included in the model is given.

The results show that the improvement in the Pearson and concordance correlation coefficients when $r_{ij}$ was included in the model was mainly for women who had more overdispersed cycles, resulting in better forecast predictions, and narrower corresponding credible intervals.

Finally, to evaluate the one-step-ahead point forecast prediction we generated prediction using a test set comprised of 1,029 women, each of whom had at least 3 repeated measurements. The results are shown in Fig. 4.

**Table 4:** Evaluation of one-step-ahead forecast prediction based on root mean square error (RMSE), concordance correlation (CCC), Pearson correlation (r), and accuracy ($\text{ C}){{\text{ b }}}$) coefficients between the predict and observed values of a new group with *N* women whom have $n_i$ observed cycles.

N	$n_i$	RMSE	CCC			r	$\text{ C}_{\text{ b }}$
			Est	Lower	Upper		
1029	3	5.2349	0.2213	0.1825	0.2610	0.2953	0.7490
760	4	5.3515	0.2254	0.1710	0.2784	0.2800	0.8048
603	5	5.4332	0.2078	0.1374	0.2760	0.2281	0.9108
434	6	5.5019	0.2102	0.1221	0.2951	0.2182	0.9634
324	7	5.6496	0.2069	0.1015	0.3078	0.2089	0.9905
248	8	6.3264	0.1047	− 0.0190	0.2252	0.1055	0.9928
199	9	6.1774	0.0778	− 0.0602	0.2129	0.0786	0.9901
160	10	5.1351	0.2632	0.1132	0.4015	0.2633	0.9998
124	11	5.1964	0.1421	− 0.0335	0.3093	0.1428	0.9954
99	12	4.8562	0.2713	0.0801	0.4433	0.2726	0.9953
78	13	4.6067	0.1970	− 0.0185	0.3951	0.2028	0.9716

As there are not the same number of repeated measurements for each woman, this makes the forecasting prediction evaluation difficult as the number of women who drop out of the test set increases over time. With this in mind, we found that RMSE values could be two times higher than those presented in Table 1, suggesting that these models are not working well for some women in the test group. The same conclusion is evident when considering the CCC and Pearson correlation coefficients. As the CCC can be written as $\text{ CCC } = \text{ r } \times \text{ C}_{\text{ b }}$, where *r* represents a measure of precision and $\text{ C}_{\text{ b }}$ a measure of accuracy^32^, we can conclude that our model has high accuracy, with the potential to increase as the number of women with repeated measurements increases. The lower precision reported for the test set suggests that the explanatory variables used in the model may not be enough to explain the variability in the data. Including additional variables such as those that capture information on polycystic ovary presence, daily diet, country of origin,may improve model forecasts in general.

## Limitations

The limitation of this study is that it is based on observational data which depends on users logging their information on the app. As a consequence, the models proposed are not intended to elucidate the causal pathway of reported symptoms on cycle length.

## Conclusion

State-space models, incorporating a probability $\pi$ as a random effect at the subject level in the random walk component. are a valuable approach for predicting menstrual cycle length. They could be used to support female athlete wellness and optimize performance. For this reason a random walk with an overdispersion parameter and an MA(1) model was selected to describe the complex dynamics of menstrual cycle length over time, which resulted in high values of CCC and Pearson correlation between observed and fitted values. Moreover, the importance of incorporating an overdispersion parameter to capture the variability of non-standard cycles was demonstrated. The data suggested that $26.36\%$ $[23.68\%,29.17\%]$ of cycles are overdispersed. The random walk standard deviation for a non-overdispersed cycle is $\sigma _{\eta }=1.0417$ [0.9971, 1.0875] days which increased to $\sigma _{w} = 4.7803$ [4.5738, 5.0007] days for non-standard cycles.

We also found that reporting injury, stomach cramps, tender breasts, and flow amount had a significant effect on menstrual cycle length amongst female athletes using the FitrWoman app. Although accurate forecast predictions are reported, improvements in the variables collected and enhancements to the model are still needed, such as considering a random effect for the moving-average coefficient $\theta _{0}$, to improve forecast precision.

## Methods

### Data characteristics

**Figure 7:**
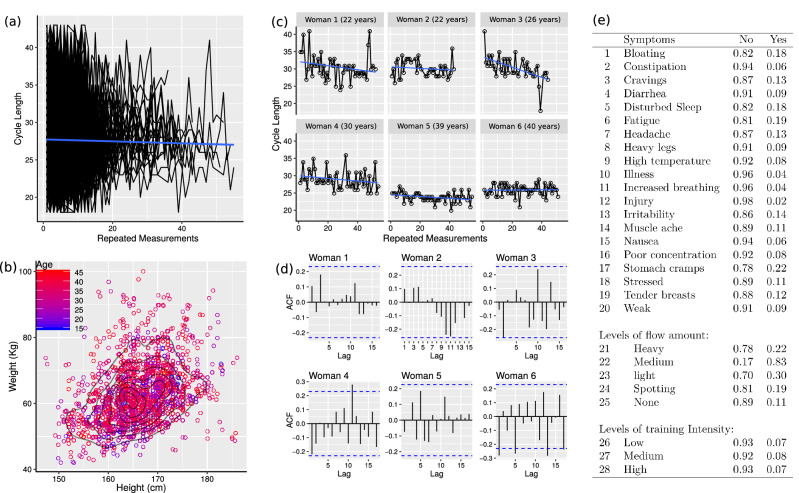
(**a**) Individual profiles of 2125 women over time; (**b**) Bivariate density plot of weight and height given age; (**c**) Individual profiles for a sample of six women with linear trend superimposed; (**d**) autocorrelation plot; (**e**) Proportion of symptoms reported, where the label “Yes” is related with “the event was reported at least one time” and “No” otherwise.

The sample was comprised of female athletes using the FitrWoman app^33^ who had given their consent for the use of their data for research purposes. The sample size contains data on 16,524 cycles collected from 2,125 women (Fig. 7a), whose mean (sd) age was 34.38 (7.05) years (range 18 to 47 years); mean (sd) weight 62.75 (9.16) Kg (range 42.18 to 100.23 Kg); mean (sd) height 165.88 (6.89) cm (range 152.4 to 186.0 cm); with several repeated measurements per woman ranging from 4 to 53 cycles. There was approximately 60% of information missing for height and weight where the 95% quantile of the sample distribution, based on 893 women, was between 153.0 and 180.0 cm for height and 48.3 and 86.11 Kg for weight. A bivariate density plot for weight and height given age is shown in Fig. 7b in order to visualise the relationship between anthropometry and age in the sample.

Menstrual cycle length is assumed to be normally distributed as the data represent standard cycles^15^, where the shortest cycle length record was 18 days and the longest was 43 days. The sample mean and variances are 27.62 and 3.51 days, respectively. As some women contributed more than one sequence to the database, we decided to consider only the first sequence available because we don’t know the reasons that caused this temporary dropout. The inclusion of the following sequences might bias the analysis, as also discussed by Bortot et al. (2010)^16^.

Figure 7c shows profiles for six women with a blue line representing a fitted mixed-effects linear regression model. It can be observed that the inclusion of a random intercept and slope plays an essential role as each woman’s cycle can be affected by different non-observed explanatory variables. However, the conditional $R^2$ was equal to 0.40, implying that the linear mixed-effects regression is a good approximation for some profiles, but not for all of them, differing from the results presented by Bull et al. (2019)^2^, who used a simple linear regression model and obtained an $R^2=0.99$. This may have happened because the number of linear profiles observed by Bull et al. (2019)^2^ is suppressing the non-linear profiles in their sample. It is clear, based on our sample, that each woman’s specific trend must be accounted for in terms of their within-subject temporal dependence and the between subject variability across women.

Figure 7c,d show that for some women a short cycle can be followed by a long cycle and vice-versa, suggesting the need for a moving-average model. Furthermore, Fig. 7d shows that cycle length for some women has a positive autocorrelation. In contrast, others have a negative autocorrelation suggesting the need for an autoregressive moving-average model incorporating individual random effects for the autocorrelation and the moving-average coefficients. Finally, Fig. 7e shows a table containing the reported proportion of reported symptoms, where in most cases symptoms did not happen or were not reported.

As a consequence of possible missing data due to non reporting of symptoms, the effect of symptoms on cycle length may be biased towards the null hypothesis of no association between symptom and cycle length (i.e. a type II error). Despite this possible bias and loss in power, the *p* values obtained from statistical methods fitted to data subject to random error or misclassification are still valid^34–36^.

### Statistical analysis

Let $Y_{ij}$ be a random variable, representing the length of menstrual cycle, where $y_{ij}$ represents the observed cycle length for the *i*-th woman, $i=1,2,\ldots ,I$ for her *j*-th menstrual cycle where $j=1,2,\ldots , J_{i}$. The main objective is to derive the one-step ahead predictive distribution given by

1 $$\begin{aligned} F_{iJ_{i}+1}\left( Y_{i,J_{i}+1}\right) = P\left( Y_{i,J_{i}+1} \le y_{i,J_{i}+1}|y_{i1}, y_{i2} \ldots , y_{iJ} \right) . \end{aligned}$$

Consequently, we are interested in evaluating $F_{i,J_{i}+1}\left( Y_{i,J_{i}+1}\right)$ under a parsimonious parametric model, that is,

$$\begin{aligned} P\left( Y_{i,J_{i}+1} \le y_{i,J_{i}+1}|y_{i1}, y_{i2} \ldots , y_{iJ} \right) = M_{i,J_{i}+1}\left( y_{i,J_{i}+1}| y_{i1}, y_{i2} \ldots , y_{iJ}, \varvec{\Theta }^{T}\right) , \end{aligned}$$

where $M_{i,J_{i}+1}\left( y_{i,J_{i}+1}\right)$ is fully specified and $\varvec{\Theta }$ is a vector of unknown fixed-effect and variance components parameters. In order to accommodate the within-subject temporal correlation between repeated measures and the between-subject variability a random walk state-space model and mixed-effects state-space model was used, incorporating a Bayesian approach for process forecasting to predict the duration, in days, of the next menstrual cycle. Each prediction is accompanied by a corresponding interval forecast as point prediction is of limited value without an accompanying measure of uncertainty^37^. We assumed that cycle length are independent and that menstrual cycles tend to decrease over time as a woman ages^15,16^. In addition, we combined the Bayesian approach and forecasting proceses to include covariates where model validation procedures were used to compare model adequacy.

#### State space models for cycle length

**Figure 8:**
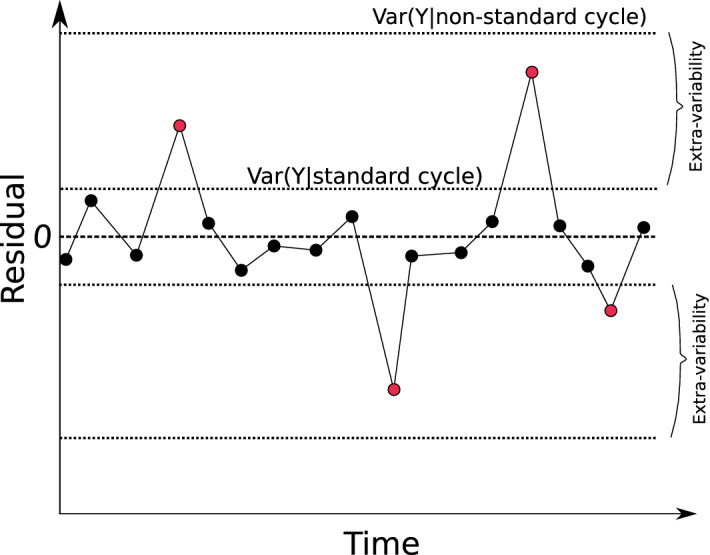
Representation of residuals ($\text{ residual}_{ij}=y_{ij}-{\hat{m}}_{ij}$) over time considering with probability $1-\pi$ of *Y* being a standard cycle (non overdispersed) and $\pi$ being a non-standard cycle (overdispersed), with $Var\left( Y|\text{ standard } \text{ cycle }\right) < Var\left( Y|\text{ non-standard } \text{ cycle }\right) .$

The state-space formulation is an attractive choice due to its flexibility to work with discrete response variables and temporal dependency amongst observations. At the same time, the mixed-effects model can be used to account for between-subject variability. As the observed event is the difference, in days, between the interval from the first day of one bleeding episode up to and including the day before the next bleeding episode, observed cycle lengths can be modelled as discrete random variables. Let $Y_{ij}$ be a continuous random variable, where $y_{ij}$ is a realisation of $Y_{ij}$, which represents the observed cycle length. Furthermore, let $O_{ij}$ be a discrete random variable, where $o_{ij}$ is a realisation of $O_{ij}$ which represents the cycle length in days as a continuous process, that is, $y_{ij} = o_{ij} + \varepsilon _{ij}$. As we have no way to estimate the error term $\varepsilon _{ij}$ (observation process), we assume that $o_{ij} = \lfloor y_{ij} \rceil$ is a good approximation for $y_{ij}$, where $\lfloor . \rceil$ indicates rounding. Thus, the true non-observed continuous cycle length $y_{ij}$ can be generated by the random walk state-space model:

2 $$\begin{aligned} y_{ij}&= m_{ij} +\gamma _{ij} + c_{ij}+r_{ij},\nonumber \\ m_{ij}&= m_{i,j-1} + \eta _{ij}, \text{ with } \eta _{ij} \sim N(0,\sigma ^{2}_{\eta }), \nonumber \\ \gamma _{ij}&= \phi \gamma _{i,j-1} + \theta \epsilon _{i,j-1}+\epsilon _{ij}, \text{ with } \epsilon _{ij} \sim N(0,\sigma ^{2}_{\epsilon }), \nonumber \\ c_{ij}&= \sum _{k=1}^{K} \alpha _{k}C_{ijk} \nonumber \\ r_{ij}&= \lambda _{ij}w_{ij}, w_{ij} \sim N\left( 0,\sigma _{w}^{2}\right) , \lambda _{ij} \sim \text{ Bernoulli }\left( \pi \right) , \pi \sim \text{ Uniform }\left( 0,1\right) \end{aligned}$$

where $y_{ij}$ is the menstrual cycle length for the *i*-th woman at *j*-th cycle; $m_{it}$ is a random walk model that allows an individual trend in the series with $\eta _{ij}$ assumed to be normally distributed with mean 0 and variance $\sigma ^2_{\eta }$. We assumed an ARMA(1,1) model for $\gamma _{it}$, where $\phi$ is the autoregressive parameter; $\theta$ is the moving average parameter; and $\epsilon _{ij}$ is assumed to be normally distributed with mean 0 and variance $\sigma _{\epsilon }^ {2}$ (process error). Furthermore, $c_{ij}$ captures the information provided by additional symptoms predictors ($C_{ij}$) that may have useful roles in understanding and forecasting cycle length, where $\alpha _{k}$ represents the *k*-th fixed effect parameter. Finally, $r_{ij}$ is a random effect term used to account for extra-variability (overdispersion) of some menstrual cycle lengths measured on *i*-th woman at cycle *j*, which could be classified as outliers. Consequently, under model (2), $y_{ij}$ has probability $\pi$ of being an overdispersed menstrual cycle (non-standard) for the *j*-th cycle measured on *i*-th woman, where its additional magnitude is given by $r_{ij}$ (Fig. 8).

In this way, $m_{ij}$ can be interpreted as the trend for a standard cycle. In contrast, $m_{ij}+r_{ij}$ can be interpreted as the trend for a non-standard cycle, where $r_{ij}$ is an overdispersion parameter at the subject level for measures which induce extra-variability, as discussed in^38^ when modelling the reported number of cases of COVID-19 where the inclusion of $r_{ij}$ allowed for the flexible modelling approach needed.

The state-space representation of the model (2) using the definition described by Brockwell & Davis (2002)^39^ is given by

3 $$\begin{aligned} y_{ij}&= m_{ij} +\gamma _{ij} + \theta x_{ij} + c_{ij}+r_{ij}, \nonumber \\ m_{ij}&= m_{i,j-1} + \eta _{ij}, \text{ with } \eta _{ij} \sim N(0,\sigma ^{2}_{\eta }), \nonumber \\ \gamma _{ij}&= \phi \gamma _{i,j-1} +\epsilon _{ij}, \text{ with } \epsilon _{ij} \sim N(0,\sigma ^{2}_{\epsilon }), \nonumber \\ x_{ij}&= \epsilon _{i,j-1}, \nonumber \\ c_{ij}&= \sum _{k=1}^{K} \alpha _{k}C_{ijk} \nonumber \\ r_{ij}&= \lambda _{ij}w_{ij}, w_{ij} \sim N\left( 0,\sigma _{w}^{2}\right) , \lambda _{ij} \sim \text{ Bernoulli }\left( \pi \right) , \pi \sim \text{ Uniform }\left( 0,1\right) \end{aligned}$$

with initial value $m_{i1} \sim N\left( \beta _{0}, \sigma _{\eta }^{2}\right)$ for the local level model and $\gamma _{ij}=\sum _{t=0}^{j-1}\phi ^{t}\epsilon _{i,j-t}$, with $j\ge 1$. The linear Gaussian state-space model defined by equation 3 are generated efficiently using the Kalman filter recursions^40^.

Fitting a separate linear regression for each woman will result in a subject-specific intercept that may account for variability due to non-observed variables likely to affect their first observed menstrual cycle. In contrast, a mixed model incorporating random slopes assumes that each woman has a different menstrual cycle length trend relative to her age. To verify if the random walk model proposed has the necessary flexibility to capture differing trends, it was compared to a linear mixed-effects model^16^. In that case, $m_{ij} = \beta _0 + b_{0i}+ \left( \beta _{1} + b_{1i}\right) Age_{ij}$, where $\beta _0$ and $\beta _1$ are the (marginal) intercept and slope, respectively; $b_{0i}$ and $b_{1i}$ are the random effects for the intercept and slope for the *i*-th woman at $Age_{ij}$, respectively, where it is assumed that

$$\begin{aligned} \varvec{b}_{i} = \left[ \begin{array}{c} b_{0i} \\ b_{1i} \end{array}\right] \sim N_{2} \left( \left[ \begin{array}{c} 0 \\ 0 \end{array}\right] , \varvec{G} = \left[ \begin{array}{cc} \sigma ^2_{b_{0}} &{} \sigma _{b_{01}} \\ \sigma _{b_{01}} &{} \sigma ^2_{b_{1}} \end{array}\right] \right) , \end{aligned}$$

and $Age_{ij}$ represents a woman’s age.

#### Bayesian implementation and choice of prior distribution

A Bayesian analysis combines information from observed data with prior distribution for the model’s parameters in order to generate a posterior distribution. In this analysis the inverse-gamma$(\kappa ,\kappa )$ is a natural candidate for the prior distributions and are often used for random walk state-space models and variance components of mixed effect models. Such a choice of prior is attractive as it can be considered as non-informative within the conditionally conjugate family, when $\kappa$ is set to a low value such as $0.1^3$:

$$\begin{aligned} \sigma _{\epsilon }^{-2},\sigma _{\eta }^{-2}, \sigma _{w}^{-2},\sigma _{b_{0}}^{-2},\sigma _{b_{1}}^{-2},\sigma _{\phi }^{-2},\sigma _{\theta }^{-2},\sigma _{\beta }^{-2}, \sigma _{ar}^{-2}\sim \text{ Gamma }\left( 0.1^3,0.1^3\right) . \end{aligned}$$

A likelihood ratio test was used to test whether the presence of correlations between the random effects in these models played a crucial role. Based on a 95% credible interval for the variance component $\sigma _{b01}$ for the proposed mixed-effects model there was sufficient evidence that the random effects are plausibly mutually independent and a term to capture the correlation structure between the intercept and slope could be removed from the model.

The choice of Prior distribution for fixed effect parameters is given by $\beta _{0} \sim N\left( \mu _{\beta _{0}}, \sigma ^{2}_{\beta }\right)$, with $\mu _{\beta _{0}} \sim \text{ Uniform }\left( 24,32\right)$; $\beta _{1} \sim N\left( \mu _{\beta _{1}}, \sigma ^{2}_{\beta }\right)$, with $\mu _{\beta _{1}} \sim \text{ Uniform }\left( -2,2\right)$; $\phi _{0},\theta _{0} \sim {\mathcal {N}}\left( \mu _{ar},\sigma ^2_{ar}\right)$, with $\mu _{ar} \sim {\mathcal {N}}\left( 0,100\right)$; and we assumed $\alpha _{k} \sim {\mathcal {N}}\left( 0, 100\right)$, which is a vague normal density prior. All assumptions were checked to make sure that results were not sensitive to specific choices of prior parameters.

#### Model selection procedure

The model selection procedure used to compare candidate models involved a balance between forecast accuracy and the Bayesian Information Criterion (BIC). Forecast accuracy was calculated based on RMSE, CCC and Pearson Correlation Coefficient while the BIC was calculated using the following formulation^41^:

$$\begin{aligned} \text{ BIC } = \left( N-p\right) \log \left( \frac{N\sigma ^2_{\epsilon }}{N-p}\right) +p\log \left[ \frac{\left( \sum _{i=1}^{I}\sum _{j=1}^{J_{i}}y_{ij}^2\right) - N\sigma ^2_{\epsilon }}{p}\right] \end{aligned}$$

where *N* is the total number of observations; and *p* is the number of parameters estimated by the model. The procedure was split into three steps namely the time trend component, the autocorrelation component, and an additional linear predictor as a function of available explanatory variables.

**Figure 9:**
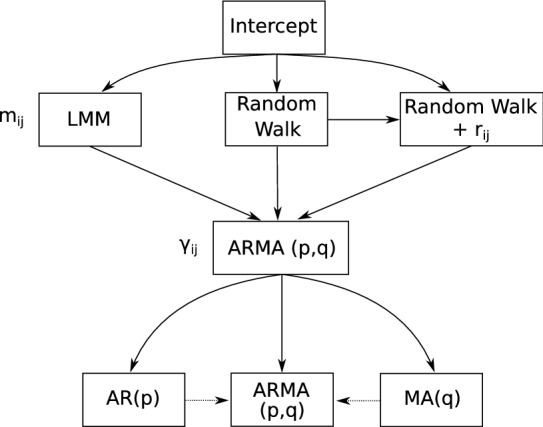
Stages 1 and 2 of the model selection procedure. LMM: linear mixed effect model; $r_{ij}$: overdispersion parameter at observational level; ARMA(*p*, *q*): Autoregressive moving average model of order *p* and *q*.

The first step was to account for a possible trend, by identifying the most appropriate error structure for the model, which in our case consisted of a comparison of a random walk model or a linear mixed effect model (Fig. 9). The second step involved the inclusion of temporal dependence among observations, as evident in some women in the sample, where an ARMA model was considered as shown in the Fig. 9. The third and final step involved the inclusion of explanatory variables to account for their (possible) relationship with cycle length. This was achieved using the posterior distribution on the parameter $\alpha _k$ to select all those variables that did not have the null value for their parameter contained in their corresponding 95% credibility interval.

**Figure 10:**
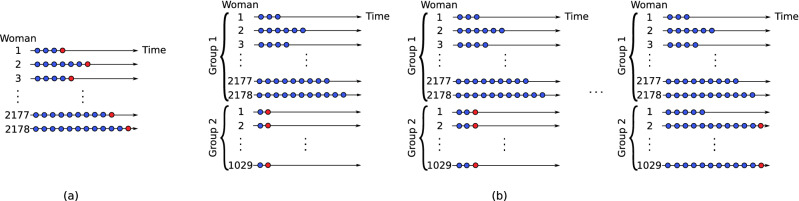
(**a**) One-step-ahead procedure for evaluation of the forecasting accuracy of training data; (**b**) time series cross validation for the test data as a function of cycle length. Training and test data are represented by blue and red circles respectively.

A novel use of train and test set data was used to validate model performance and to estimate the one-step ahead forecasting prediction accuracy as a function of the number of cycles reported. The complete sample of 2125 was used for model validation by treating the last observed cycle length as test data. The procedure is illustrated in Fig. 10 where the last observed cycle length (red dot) is ‘held back’ as test data and the remaining data (blue dots) were used as training data. The forecast performance was calculated using the RMSE and CCC between the observed and predicted cycle lengths and used jointly with the BIC criteria in the model selection process.

Once the model was selected, it was then sequentially tested using i) the complete data as training data and ii) a random sample of 1029 (approximately half the complete data) as test data. As the number of cycles reported varied from 2 to 12, one-step ahead forecasting prediction accuracy was calculated for each of these scenarios by treating the last observed cycle in each scenario as test data. As the number of athletes in the test set decreased with increasing reported cycle lengths, individuals that had fewer observed cycle lengths for the cycle length scenario under consideration were included in the training set to account for this attrition.

The forecast error for an observed value and its forecast was computed as

$$\begin{aligned} \epsilon _{i,J_i+h}=y_{i,J_i+h}-{\hat{y}}_{i,J_i+h|J_i} \end{aligned}$$

where the training data are given by $\left\{ y_{i1},y_{i2},\ldots , y_{iJ_i}\right\}$ and the test data by $\left\{ y_{i,J_i+1}\right\}$ (i.e. one-step ahead prediction for each woman), see Fig. 10. The forecast accuracy was measured by the root mean square error (RMSE), concordance correlation coefficient^32^, and the Pearson correlation coefficient between the observed response in the test data and corresponding predicted cycle length value.

#### Posterior computation

Markov Chain Monte Carlo (MCMC) was used to generate samples from the posterior distribution for the random walk and mixed-effects state-space models using a Gibbs sampler algorithm^40^, as this approach is widely used to obtain parameter estimates from a posterior distribution. The convergence of the MCMC algorithm was checked by multiple comparisons of MCMC chains with different starting points. The normality assumptions were checked using suitable residual plots and quantile-quantile plots with simulate envelopes^42^. The one-ahead predictive distribution of $F_{i,J_{i}+1}\left( Y_{i,J_{i}+1}\right)$ was derived through draws from the posterior distribution. Consequently, the $\kappa$-step ahead predictive distribution was obtained by running the Kalman filter sequentially. All analysis were implemented in R including runjags^43^, coda^44^, hnp^42^, and ggplot2^45^ packages.

### Ethics approval

This publication has emanated from research supported in part by a research grant from Science Foundation Ireland (SFI) under Grant Number SFI/12/RC/2289, co-funded by the European Regional Development Fund in partnership with Orreco. All methods were carried out in accordance with relevant guidelines and regulation. In particular the data that support this study were made available by ORRECO. Upon first use, all FitrWoman app users provide informed consent by agreeing to their anonymised data being used with third parties for research purposes. However, restrictions apply to the availability of these data used under license for the current study. In order to use the Fitrwoman app each participant must agree to the following conditions: Without prejudice to the foregoing, ORRECO shall have an exclusive, royalty free, perpetual licence to use and retain the User Data and all other information arising from the provision of the Services:- (i) for research purposes, (ii) in order to improve the standard of service provided by ORRECO in the future; (iii) in order to validate ORRECO’s proprietary algorithms or intervention programmes; (iv) to analyse and report anonymously on patterns in User Data by reference to their age, sex, ethnicity, discipline, field, training schedule, performance, results or such other data sets as ORRECO may decide; and (v) in order to develop similar or new services, provided that in each case the identity of the User and any personal data comprised within the User Data shall be kept, removed or anonymised. Anonymised data shall be sent to third party processors to be analysed to uncover patterns and trends and to further sports science research. The FitrWoman app is compliant with the General Data Protection Regulation laws (GDPR 2016/679). All experimental protocols and ethical use of data were approved by the ethics committee of the Insight Centre for Data Analytics, National University of Ireland Galway, Ireland.

## Supplementary Information

Supplementary Information 1.

Supplementary Information 2.

Supplementary Information 3.
