# Supplementary figures and images for: Modelling menstrual cycle length in athletes using state-space models
Source: Sci Rep. 2021 Aug 20;11:16972. doi: 10.1038/s41598-021-95960-1 (PMC8379295; doi:10.1038/s41598-021-95960-1)

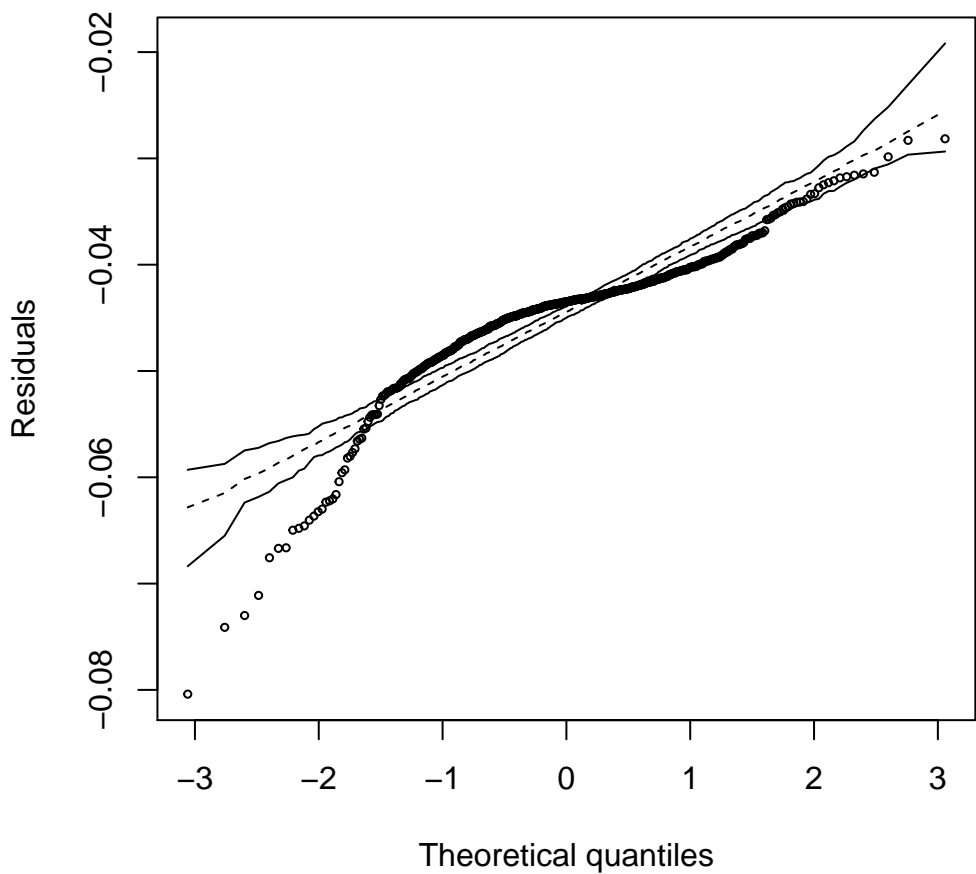

Supplement: Supplementary file 1 — Supplementary Information 1. [file 41598_2021_95960_MOESM1_ESM.pdf]

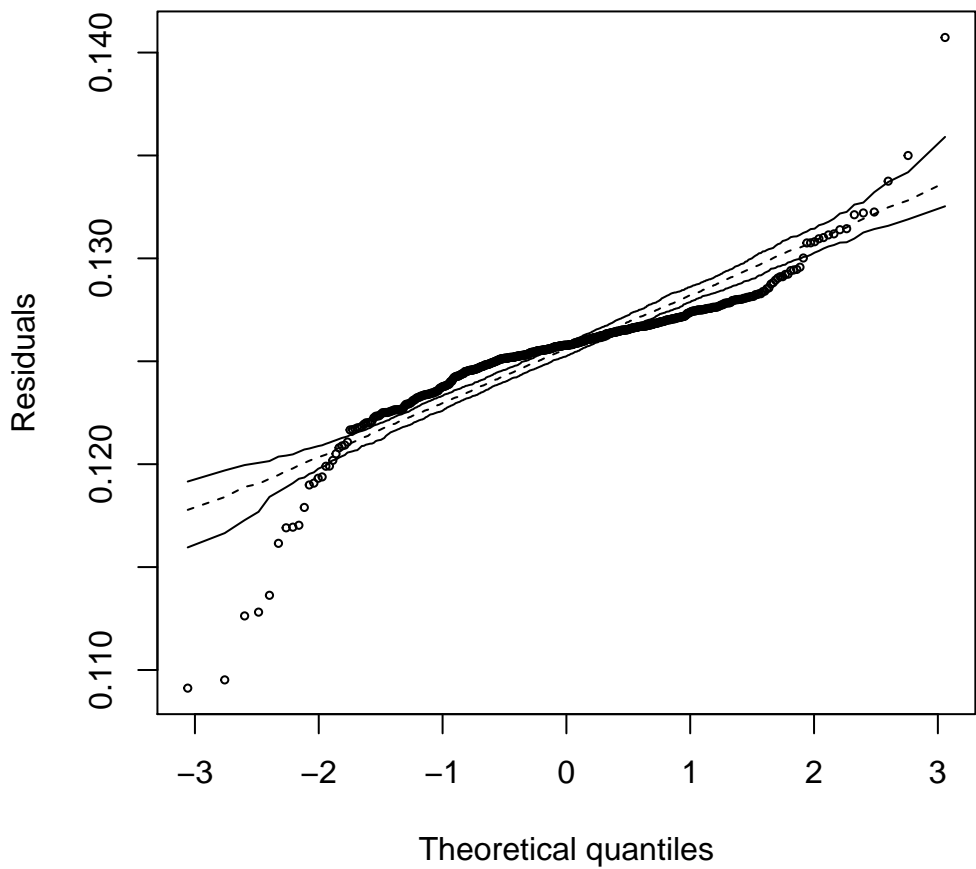

Supplement: Supplementary file 2 — Supplementary Information 2. [file 41598_2021_95960_MOESM2_ESM.pdf]

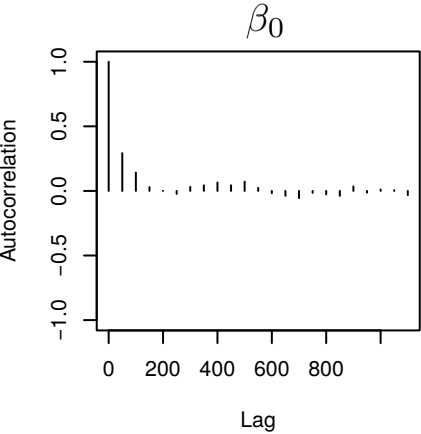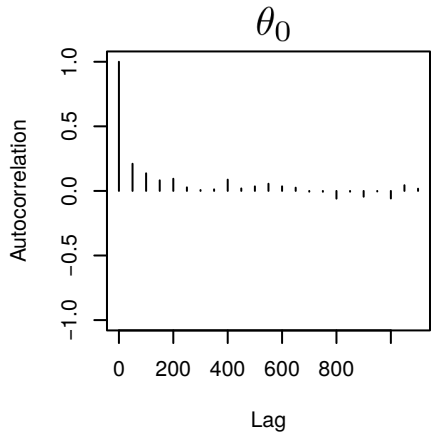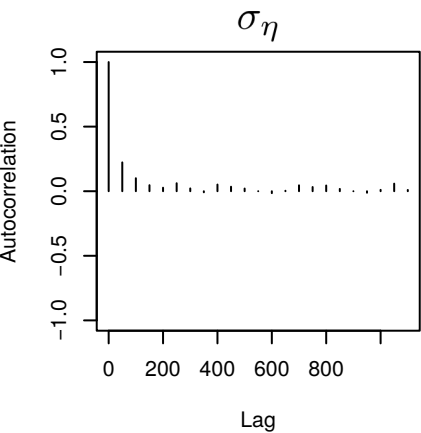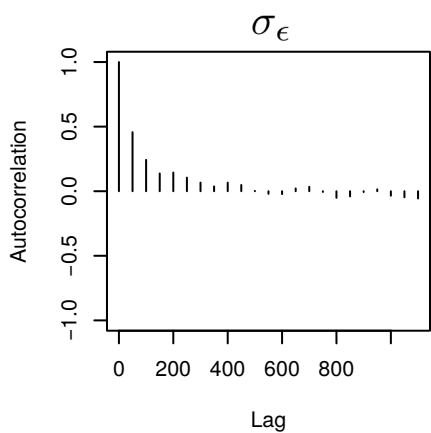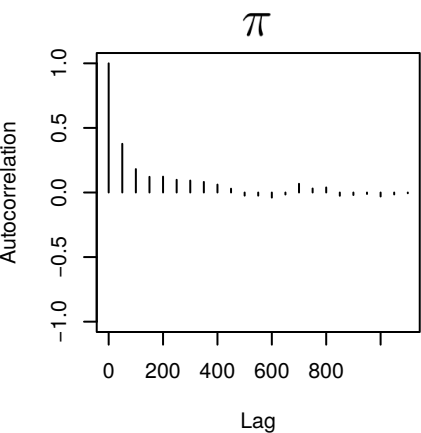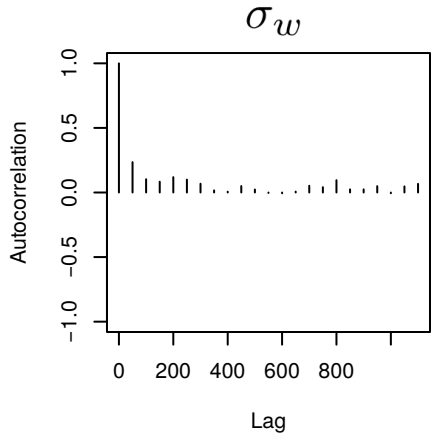

Supplement: Supplementary file 3 — Supplementary Information 3. [file 41598_2021_95960_MOESM3_ESM.pdf]
